# Chronic IL-6 Administration Desensitizes IL-6 Response in Liver, Causes Hyperleptinemia and Aggravates Steatosis in Diet-Induced-Obese Mice

**DOI:** 10.1371/journal.pone.0157956

**Published:** 2016-06-22

**Authors:** Ana Luisa Gavito, Dolores Bautista, Juan Suarez, Samir Badran, Rocío Arco, Francisco Javier Pavón, Antonia Serrano, Patricia Rivera, Juan Decara, Antonio Luis Cuesta, Fernando Rodríguez-de-Fonseca, Elena Baixeras

**Affiliations:** 1 Laboratorio de Investigación, IBIMA, Hospital Regional Universitario de Málaga, 29010, Málaga, Spain; 2 Centro de Investigación Biomédica en Red de Fisiopatología de la Obesidad y Nutrición (CIBERobn), Instituto de Salud Carlos III (ISCIII), 28029, Madrid, Spain; 3 Unidad de Gestión Clínica de Salud Mental, Hospital Regional Universitario de Málaga, 29010, Málaga Spain; 4 Unidad de Gestión Clínica de Anatomía Patológica, Hospital Regional Universitario de Málaga, 29010, Málaga, Spain; 5 University College South Denmark, 6705, Esbjerg, Denmark; 6 Danish Diabetes Academy, Department of Biomedical Sciences, Panum Institute, University of Copenhagen, Copenhagen, Denmark; University of Navarra School of Medicine and Center for Applied Medical Research (CIMA), SPAIN

## Abstract

High-fat diet-induced obesity (DIO) is associated with fatty liver and elevated IL-6 circulating levels. IL-6 administration in rodents has yielded contradictory results regarding its effects on steatosis progression. In some models of fatty liver disease, high doses of human IL-6 ameliorate the liver steatosis, whereas restoration of IL-6 in DIO IL-6^-/-^ mice up-regulates hepatic lipogenic enzymes and aggravates steatosis. We further examined the effects of chronic low doses of murine IL-6 on hepatic lipid metabolism in WT mice in DIO. IL-6 was delivered twice daily in C57BL/6J DIO mice for 15 days. The status and expression of IL-6-signalling mediators and targets were investigated in relation to the steatosis and lipid content in blood and in liver. IL-6 administration in DIO mice markedly raised circulating levels of lipids, glucose and leptin, elevated fat liver content and aggravated steatosis. Under IL-6 treatment there was hepatic Stat3 activation and increased gene expression of *Socs3* and *Tnf-alpha* whereas the gene expression of endogenous *IL-6*, *IL-6-receptor*, *Stat3*, *Cpt1* and the enzymes involved in lipogenesis was suppressed. These data further implicate IL-6 in fatty liver disease modulation in the context of DIO, and indicate that continuous stimulation with IL-6 attenuates the IL-6-receptor response, which is associated with high serum levels of leptin, glucose and lipids, the lowering levels of lipogenic and Cpt1 hepatic enzymes and with increased *Tnf-alpha* hepatic expression, a scenario evoking that observed in IL-6^-/-^ mice exposed to DIO and in obese Zucker rats.

## Introduction

Increased plasma IL-6 levels are normally associated with obesity and fatty liver disease [1–4], but the involvement of IL-6 in the molecular mechanisms underlying the pathogenesis of lipid and carbohydrate metabolism is not fully understood [5–7]. Indeed, it is a subject of excited debate in the literature [8–13]. Regarding hepatic lipid metabolism, evidence suggests that IL-6 affects the degradation as well as synthesis of fatty acids [10, 12, 14–18]. The fact that cytokines, such as IL-6, are subjected to a rigorous signalling feedback control and that some of them can share their receptor chains and signalling pathways may complicate the interpretation of the role of a cytokine in a given scenario [19, 20].

Previous studies have shown a beneficial role of IL-6 against several models of fatty liver, including alcohol liver disease [10, 21–23]. Moreover, the lack of IL-6 predisposes to liver steatosis, thus reinforcing *a priori* the idea that IL-6 contributes to alleviating steatosis [12, 22, 24]. These beneficial effects were attributed in part to the ability of IL-6 to mediate mitochondrial *beta*-oxidation of fatty acids, increase the hepatic export of triglycerides and cholesterol, and to its antioxidant, anti-apoptotic effects on hepatocytes [10, 17, 18, 21].

From the above, although one could infer that IL-6 has a protective role in fatty liver diseases, it is nevertheless striking that both alcoholic and non-alcoholic fatty liver diseases (NAFLD) are associated with elevated serum levels of IL-6 as well as increased blood concentrations of lipids and glucose [1, 25, 26]. Likewise, high doses of IL-6 increase blood levels of lipids and glucose [27–29]. Indeed, in chronic inflammatory autoimmune diseases such as rheumatoid arthritis, in which the production of IL-6 is deregulated, the circulating lipid levels (total cholesterol and triglycerides) are increased while blockade of IL-6 decreases their levels [30]. In a recent study we showed that the chronic replacement of IL-6 with physiological doses in IL-6^-/-^ mice seriously aggravates the steatosis induced by a high-fat diet [12]. This effect was accompanied by the up-regulation of the lipogenic enzymes, thereby possibly contributing to fatty acid accumulation through *de novo* production in the liver of these mice [12]. Thus, the question also arises concerning whether the higher levels of lipogenic enzymes in the liver are related to high levels of circulating IL-6.

IL-6 acts via the gp80/gp130 complex which is expressed mainly in leukocytes and those cells where fatty acid synthesis occurs, as adipocytes and hepatocytes [31, 32]. IL-6 binds initially to the non-signalling interleukin-6 receptor (IL-6R or gp80), which subsequently leads to the recruitment of two gp130 receptor proteins. The IL-6 receptor complex promotes activation of the signal transducer and activator of transcription 3 (Stat3) through the Jak kinase [19]. Once Stat3 is tyrosine phosphorylated (activated) it translocates as a dimer into the nucleus, where it activates specific genes [33]. Recent studies have revealed that mRNA levels of the lipogenic enzymes acetyl-CoA carboxylase (*Acac*) and fatty acid synthase (*Fasn*) are increased through the hepatic over-expression of Stat3 [34]. Interestingly, treatment of mice with IL-6 increases the levels of *Stat3* mRNA in the liver [35]. Moreover, we reported that the gene expression of the lipogenic enzymes Acac, Fas and Stearoyl-CoaA desaturase (Scd1) was no longer up-regulated by IL-6 in the presence of siRNA Stat3 in hepatocytes, therefore indicating that IL-6-mediated signalling promotes the expression of these enzymes *via* activation of Stat3 [36].

Inhibition of the Stat3 pathway can occur by two main elements: the suppressor of the cytokine signalling 3 (Socs3) protein, which acts through inhibition of Jak/Stat at the level of the IL-6 receptor in the membrane; and by the protein inhibitor of activated Stat3 (Pias3), which inhibits Stat3/DNA binding in the nucleus [20]. The mRNA for *Socs3* is rapidly induced upon IL-6 stimulation and its protein inhibits IL-6-mediated signalling in a classic feedback loop. Socs3 deficiency results in prolonged activation of Stat3 after IL-6 stimulation and, interestingly, also promotes lipogenesis, thereby leading to fat accumulation and inflammation in the liver [37, 38]. The interaction of Pias proteins with Stat factors requires tyrosine phosphorylation (activation) of the Stat proteins [39]. Thus for example, Pias3 inhibits the gene expression mediated by phosphorylated Stat3 after IL-6 stimulation [39].

In a recent study we observed that a single low dose of IL-6 up-regulated the gene expression of lipogenic enzymes in IL-6^-/-^ mice under a normal chow diet [36]. However, notably, this phenomenon was less obvious in the corresponding counterpart wild-type (WT) mice, which appeared less receptive to IL-6 treatment [36]. Interestingly, the repeated administration of human IL-6 to WT mice causes complete remission of the fatty liver diseases [10, 21, 22] whereas the replacement of IL-6 in IL6^-/-^ mice with fatty liver aggravates the steatosis [12]. These two opposing biological actions of IL-6 in WT and IL-6^-/-^ mice highlight the fact that the role of IL-6 in metabolic liver disease is not completely understood.

In this study we further explore the effects of chronic administration of IL-6 in WT mice in a situation of diet-induced obesity (DIO), a situation that already increases the endogenous circulating IL-6 levels and induces fatty liver. The results presented herein indicate that, the administration of exogenous IL-6 in WT mice worsened the steatosis. However, the expression of hepatic lipogenic enzymes remained strongly reduced. The findings suggest a mechanism for desensitizing IL-6-mediated signalling in order to abolish the continuous stimulation of the IL-6-receptor in a WT background. This phenomenon may not only have implications in the modulation of the severity of the steatosis but it may also help to understand the controversial data about the role of IL-6 in the liver.

## Material and Methods

### Animals and ethical statement

The experiments were performed on 12 week-old male mice from the C57BL/6J (SN 0664) strain (Charles River Laboratories, Barcelona, Spain). The mice were housed with a 12 hour—12 hour light/dark cycle and fed a standard chow diet *ad libitum*. Water and chow pellets were available *ad libitum* throughout the course of the present study.

Mice were split in two groups: one group fed a regular chow diet (STD; Harlam Teklad, Madison WI) for 16 weeks, and a second group fed a high-fat diet (HFD, diet-D12492; Research Diets Inc., New Brunswick, NJ, USA) for 16 weeks. After 16 weeks of HFD feeding, the mice were chronically treated with either murine recombinant IL-6 (rIL-6, Peprotech, Inc., Rocky Hill, NJ, USA) or vehicle (0.1% BSA in PBS) alone as previously described [12]. For some experiments IL-6-deficient (IL-6^-/-^) mice, strain B6.129S2-IL-6^tm1Kopf^/J (SN 2650, http://jaxmice.jax.org/strain/002650.html), were also included. Since the levels of lipogenic enzymes in liver may vary depending on the time of food intake or fasting [36], both strains were maintained in the same feeding/fasting conditions. The day of sacrifice, animals were fasted from 08:00 h. The mice were treated with the last inoculation of rIL-6 or vehicle at 12:00 h and were sacrificed 1 hour later.

All experimental procedures with animals were conducted in accordance with the Spanish Legislation (Real Decreto 53/2013, BOE, 34/-11421, 2013) in compliance with the European Community Directive 2010/63/EU regulating the use and care of laboratory animals. The protocols were approved by the Ethics Committee (Permit number: 2012-0070-A) for Animal Experiments of the University of Malaga. The animals were anaesthetized using isoflurane to minimize animal suffering before sacrificing via decapitation.

### Blood sampling, and serum biochemical and cytokine analysis

Blood sample collection was performed as previously described [12]. The IL-6 and leptin concentrations in the sera were assayed using ELISA kits specific for the mouse IL-6 (Millipore, Temecula, CA, USA), and leptin (Abcam, Cambridge, UK) according to the manufacturer’s instructions. Biochemical parameters were assessed as previously described [12].

### Histological evaluation and liver fat extraction

Liver samples embedded in paraffin were sectioned into 3 μm slices and deparaffinized in xylene followed by haematoxylin and eosin (H&E) stainning. Total fat was extracted from the liver and assessed as previously described [12, 40].

### RNA isolation, RT-qPCR analysis

Total RNA from liver sections was extracted and reverse transcribed as described [12]. The expression of the genes encoding for mouse Acc-alpha (*Acaca*), Acc-beta (*Acacb*), Fas (*Fasn*), Scd1 (*Scd1*), Cpt1 (*Cpt1*), Tnf-alpha (*TNF-alpha*) Socs3 (*Socs3*), Srebp-1(*Srebp-1*) and Lxr (*Lxr*) was measured through qPCR by using FastStart Universal SYBR Green Master (Rox) (Roche Applied Science, Mannheim, Germany). Mouse glyceraldehyde-3-phosphate dehydrogenase *(Gapdh*) and mouse beta-glucuronidase (*Gus-beta*) were used as reference genes. Specific oligonucleotides were designed at Universal Probe Library (Roche Applied Science) to amplify particular regions of the genes of interest. Specific primers for the mouse *IL-6* gene as well as the reference genes *Gapdh* and *Gus-beta* were obtained from Taqman^®^ Gene Expression Assays (Life Technologies). The gene symbols, GeneID, primer sequences and amplicon lengths, except those for *Stat3*, are described elsewhere [12, 36]. For mouse *Stat3* (Gene ID: 20848) expression the following primers were used: *mStat3* forward GTTCCTGGCACCTTGGATT and reverse CAACGTGGCATGTGACTCTT. All PCR reactions were performed using a CFX96 Real-Time PCR Detection System (Bio-Rad, Hercules, CA, USA) and Cq and efficiency value calculation for each experimental set was performed as described [12]. The calibrated normalized relative quantity values were exported from the qBase^PLUS^ software and statistically analyzed.

### Protein Extraction and Western Blot Analysis

The protein extraction and western blot analysis were performed as described [12]. Specific proteins were detected after incubation of blotted membranes with the corresponding primary antibodies: rabbit anti-Acc-alpha/beta, anti-Fas, anti-Scd1, anti-Stat3, anti-phospho-Stat3 (Tyr705) and anti-Actin antibodies (Cell Signalling Technology Inc. MA, USA). Rabbit anti-Cpt1A, anti-LepR and anti-adaptin gamma antibodies were purchased from Abcam (Cambridge, UK), anti-phospho LepR (Tyr 1077) antibody was from Millipore (Temecula, CA, USA) and anti-Socs3 and anti-Pias3 antibodies were from Santa Cruz (Biotechnology Inc., CA, USA). The specific protein bands were revealed using an anti-rabbit HRP-conjugated antibody as secondary antibody (Promega, Madison, MI, USA) followed by detection through the enhanced chemiluminescence system (Santa Cruz, Biotechnology Inc. CA, USA) as described [12]. The levels of specific proteins were normalized to actin or adaptin levels.

### Statistical analysis

All data in the graphs and tables are expressed as the mean ± standard error of the mean (SEM). The GraphPad Prism version 5.04 software was used for statistical analysis of the results (GraphPad Software Inc., San Diego, CA, USA). The significance of the differences between groups (diet) was evaluated using one-way analysis of variance (ANOVA), followed by a *post hoc* test for multiple comparisons (Bonferroni test). *P-*values less than 0.05 were considered statistically significant.

## Results

### 1. Chronic administration of IL-6 aggravates the steatosis and increases serum leptin levels in wild-type mice in DIO

**Fig 1:**
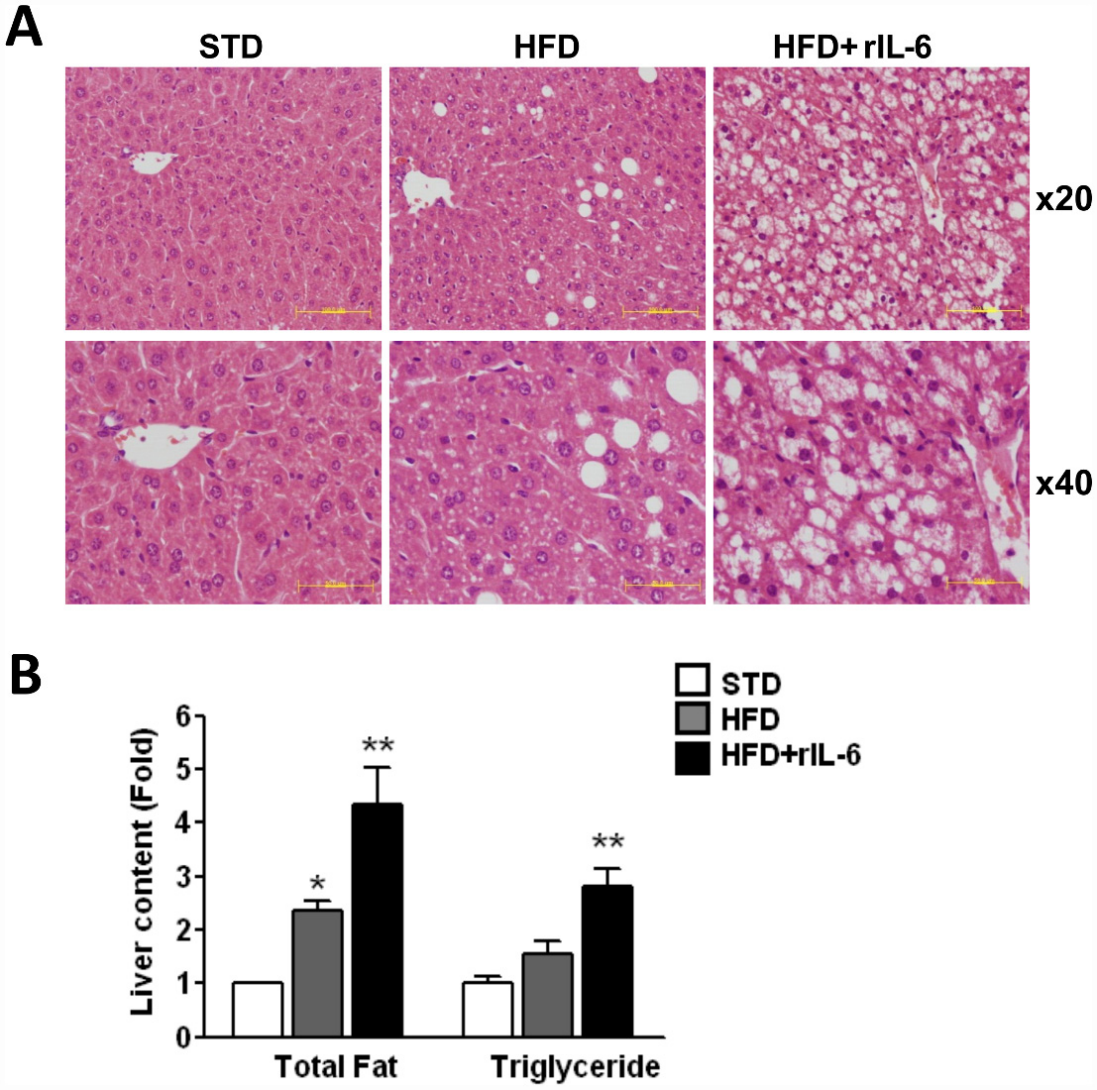
Effect of IL-6 chronic treatment on steatosis and fat liver content in WT mice fed a HFD. **(A)** Representative histological appearance of the liver sections (haematoxylin and eosin-stained) from mice fed a STD, HFD or HFD-treated with rIL-6 (HFD+rIL-6). The accumulation of lipid droplets is evident in the livers of HFD mice, revealing moderate steatosis in HFD samples and marked and diffuse microvesicular and macrovesicular steatosis in HFD+rIL-6 samples. Scale bars: 100 μm and 50 μm for x20 and x40 magnifications, respectively **(B)** Hepatic fat and triglyceride content in the samples from mice fed STD, HFD or HFD+rIL-6. The values are presented as the means ± SEM (n = 8 samples per group), and differences between diet conditions were evaluated using one-way ANOVA analysis. *P<0.05 and **P<0.01 denote significant differences compared with the corresponding STD-fed group.

To further examine the effects of IL-6 in NAFLD we induced steatosis in WT mice with a HFD for 16 weeks. In parallel, a group of mice were fed a STD. During this period, the group fed a STD gained 10.5 g while the group fed a HFD gained 22 g and became obese. The HFD-fed group was split in two subgroups: one subgroup was treated with saline (HFD) and the other subgroup was treated with recombinant IL-6 (HFD+rIL-6) for an additional 15 days, as described in Material and Methods. Fig 1A illustrates the liver histology of mice fed a STD, a HFD, and a HFD treated with rIL-6. Livers from mice fed a STD showed a normal parenchyma whereas those of HFD-fed mice had increased lipid content, exhibiting a moderate diffuse macrovesicular steatosis. The fatty changes were more pronounced in HFD-fed mice treated with rIL-6, resulting in a severe macro- and microvesicular steatosis, with predominant accumulation of small lipid droplets in hepatocytes. No significant inflammatory (lymphocyte infiltrates) reaction was seen. Fat storage was also measured by determining the total fat and triglyceride contents in the liver (Fig 1B). Compared with the STD group, total fat was 2.3-fold higher (*P*<0.05) in the HFD group and 4.3-fold higher (*P*<0.01) in the HFD group treated with rIL-6 (Fig 1B). Likewise, the triglyceride content tended to increase (1.5-fold) in the livers from HFD-fed mice, becoming significantly higher (2.8-fold; *P*<0.01) in livers from the HFD group receiving rIL-6 (Fig 1B). The severity of fatty liver after a HFD was also associated with significant increases in serum concentrations of cholesterol (1.5-fold, *P*<0.01) as compared with the cholesterol levels found in STD-fed mice (Table 1). Cholesterol levels were significantly higher (*P*<0.001) in the rIL-6 treated group, in which serum cholesterol concentrations were increased 2-fold (*P<*0.001), compared with the STD group (Table 1). However, serum triglyceride, HDL-cholesterol, GPT and GGT levels showed no significant changes in any of the groups.

**Table 1:** Biochemical parameters in serum.

	Cholesterol	Triglycerides	HDL	GPT	GGT
**STD**	86.25 ± 3.8	60.25 ± 10.4	21.00 ± 2.4	36.75 ± 3.8	8.25 ± 1.6
**HFD**	129.00 ± 7.6**	45.00 ±13.2	24.00 ± 2.1	40.00 ± 3.2	7.00 ± 1.2
**HFD +rIL-6**	171.00 ± 6.3***	47.00 ± 11.6	20.00 ± 1.2	36.00 ± 2.1	8.00 ± 1.2

*** *P<0*.*001 vs* STD

**P<0.*01 vs* STD

The circulating levels of glucose and the cytokines IL-6 and leptin were also measured in serum of WT and IL-6^-/-^ mice in DIO. As shown in Table 2, the basal blood glucose levels were increased (*P*<0.05) in WT DIO mice after rIL-6 treatment, as they were in IL-6^-/-^ DIO mice without any treatment (*P*<0.01) (Table 2). Increased levels of IL-6 (5-fold; *P*<0.001) and leptin (1.5-fold; *P*<0.05) were observed in the HFD-fed mice as compared with the STD group. The treatment with rIL-6 not only markedly increased the serum levels of IL-6 (8-fold; *P*<0.001) with respect to the STD group (as expected), but it also increased (3.6-fold, *P*<0.001) the circulating leptin levels. This latter observation suggested that these mice developed leptin resistance. This may seem somewhat paradoxical because hyperleptinemia and leptin resistance have been described in obese IL-6^-/-^ mice and are therefore associated with a lack of IL-6 rather than increased levels of IL-6 [13]. Indeed, samples from mice deficient for IL-6 and maintained in the same DIO conditions showed a marked increase in serum leptin levels as compared with the corresponding STD-fed control group (Table 2).

**Table 2:** Levels of IL-6, leptin and glucose in serum.

	IL-6 (pg/ml)	Leptin (pg/ml)	Glucose (mmol/l)
**WT STD**	18.22 ± 0.3	250.96 ± 10.1	135 ± 19
**WT HFD**	89.57 ± 4.5***	368.00 ± 21*	131 ± 17
**WT HFD +rIL-6**	145.35 ± 3.6***	896.84 ± 40.1***	198 ± 9.9*
**IL-6**^**-/-**^ **STD**	_	204.48 ± 9.9	230 ± 16
**IL-6**^**-/-**^ **HFD**	_	928.19 ± 1.9 ^###^	309 ± 7.64^##^

*P<0.*05 vs* WT STD

*** *P<0*.*001 vs* WT STD

^##^ *P*<0.01 *vs* IL-6^-/-^ STD

^###^ *P<0*.*001 vs* IL-6^-/-^ STD

**Fig 2:**
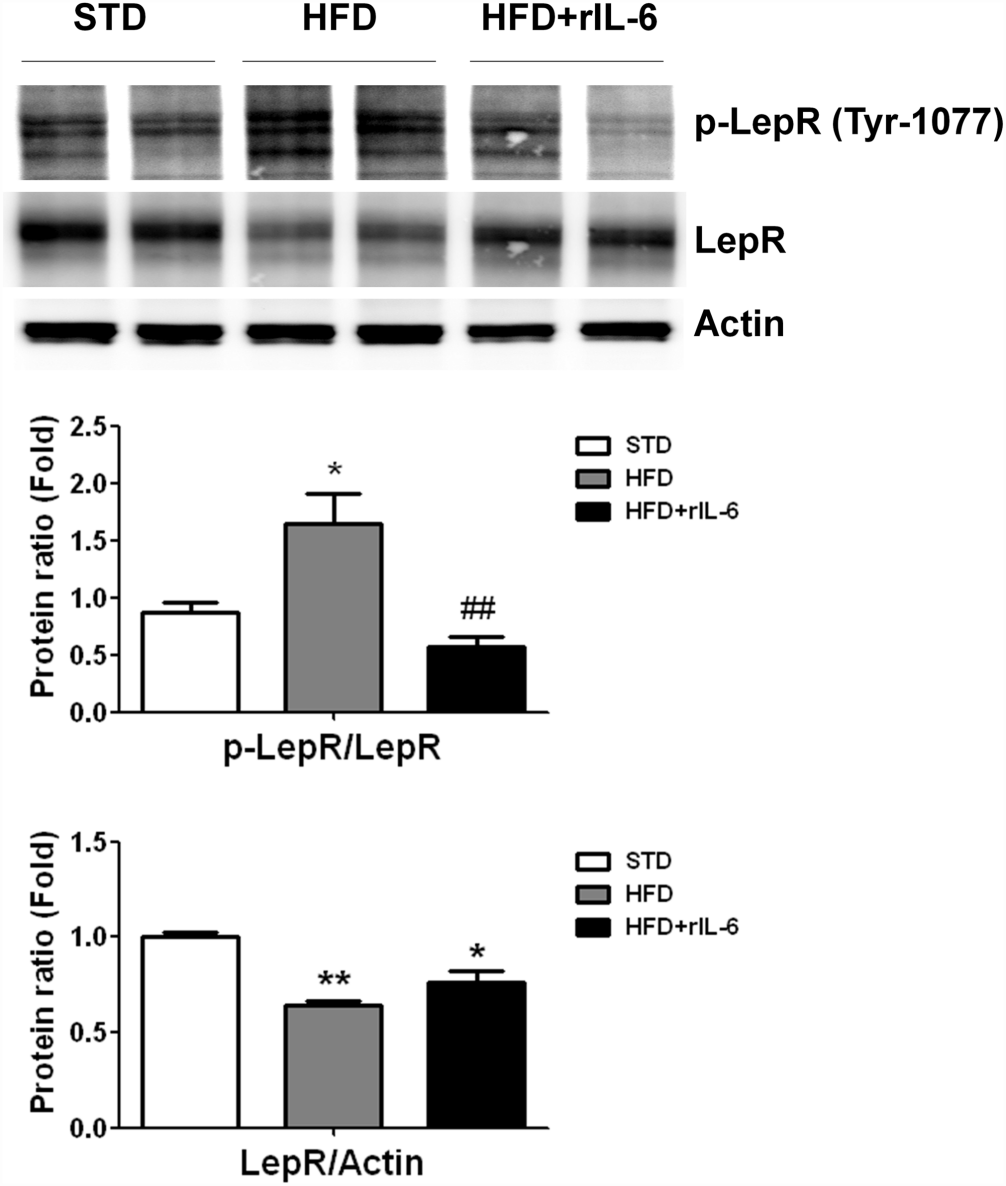
Leptin receptor (LepR) expression and Tyr (1077) phosphorylation status in liver of HFD-fed mice untreated or treated chronically with rIL-6. Representative western blot analysis for total LepR and its phosphorylated form (p-LepR) out of five samples per group (STD, HFD and HFD+rIL-6). The corresponding expression of actin is shown as loading control per lane. The ratios for LepR/actin and p-LepR/LepR determined through densitometry are shown in the histograms below. The values represent the means ± SEM. The significance of differences between groups was evaluated using one-way ANOVA. *P< 0.05 and **P< 0.01 denote differences compared with the STD group, ^##^*P*< 0.01, denotes differences compared with HFD group.

We next investigated the expression of the leptin receptor (LepR) and its tyrosine phosphorylation status (p-LepR) in the liver tissue of WT mice from the different groups. We found that LepR phosphorylation was increased in the HFD-fed group despite detecting lower total protein levels (Fig 2). This observation might reflect receptor activation and degradation after binding to ligand. The HFD-fed mice treated with rIL-6 showed reduced pLepR/LepR ratio, thus suggesting a lesser LepR activity. Given that this animal group showed high levels of circulating leptin, this last observation is compatible with a leptin resistance after rIL-6 treatment.

### 2. Chronic administration of IL-6 down-regulates the expression of the hepatic enzymes of fatty acid metabolism in the DIO wild-type mice

**Fig 3:**
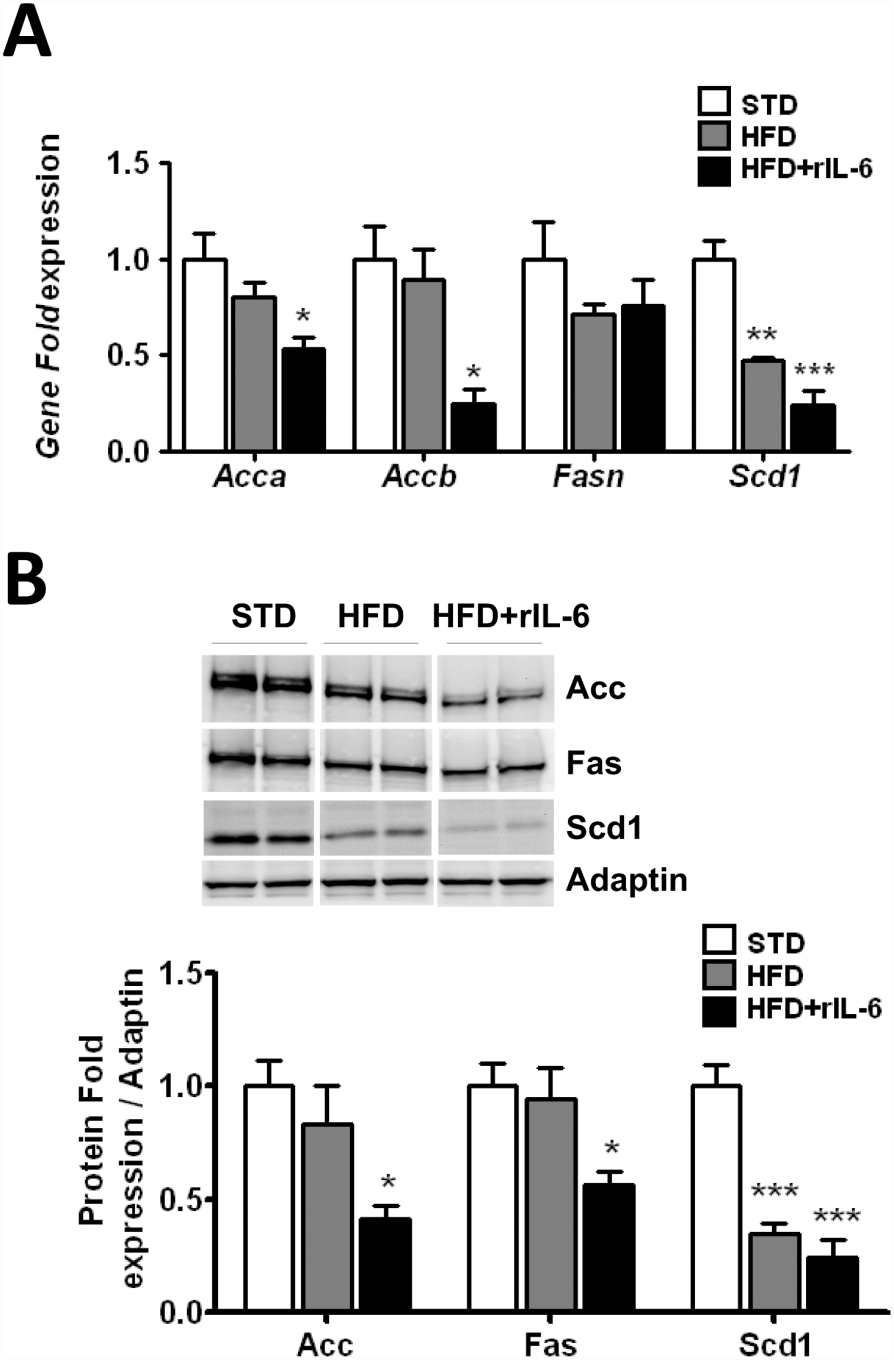
Effect of IL-6 treatment on the expression of lipogenic enzymes in the livers of WT mice fed a HFD. **(A)** The gene expression of *Acaca*, *Acacb*, *Fasn*, and *Scd1* in the livers of WT fed STD, HFD or HFD-treated chronically with rIL-6 (HFD+rIL-6) is depicted in the corresponding histogram. The gene expression was determined through qPCR analysis of the liver samples. The expression of each gene was normalized using Biogazelle’s qBase^PLUS^ software with *Gapdh* and *Gus-beta* as reference genes. The columns represent CNRQ means ± SEM (n = 8 animals per group). (**B**) Western blot analysis of the protein expression of Acc-alpha/beta, Fas, and Scd1 in liver samples from WT and mice fed a STD, HFD or HFD+rIL-6. Representative blots of each protein from two samples out of five per group are presented in the upper panel. The corresponding expression of adaptin is shown as a loading control per lane. The histograms below blots depict the levels of each Acc-alpha/beta, Fas and Scd1 protein determined through densitometry and corrected for adaptin. The values represent the means ± SEM (n = 8 samples per group). Comparisons between diet conditions for each gene/protein in A and B were analyzed using one-way ANOVA. *P<0.05, **P<0.01 and ***P<0.001 denote significant differences compared with the corresponding STD-fed group.

The results described above are in line with the situation found previously in IL-6^-/-^ mice in which the chronic restitution of IL-6 aggravated the steatosis rather than improving it [12]. In that case, the aggravation of steatosis observed after treatment with IL-6 was accompanied by up-regulation of lipogenic enzymes [12]. Here we show that WT mice fed a HFD experience no major changes regarding the hepatic *Acaca*, *Acacb* and *Fasn* levels, but a significant decrease (*P*<0.01) in *Scd1* expression as compared with the levels found in the STD condition (Fig 3A). Unlike in the IL-6^-/-^ mice, the treatment with rIL-6 in the WT mice in DIO significantly reduced (*P*<0.05) the expression of *Acaca*, *Acacb*, and the expression of *Scd1* was reduced even further (*P*<0.001) (Fig 3A). No major changes were observed, however, for *Fasn* expression in any of the diet conditions assayed, even in samples from mice treated with rIL-6 (Fig 3A). Interestingly, the gene expression profile of the lipogenic enzymes was very similar to that described previously for IL-6^-/-^ mice in DIO and untreated with rIL-6 [12]. The protein expression of Acc-alpha/beta, Fas and Scd1 was also examined through western blotting (Fig 3B). Paralleling their gene expression, exposure to a HFD did not change the expression of the hepatic Acc-alpha/beta doublet or Fas levels, except for that of Scd1 which was reduced (*P*<0.001) compared with the STD group (Fig 3B). A significant reduction (*P*<0.05) in the expression of the Acc-alpha/beta doublet and Fas enzymes was observed in samples from mice fed a HFD and treated with rIL-6, and levels of the Scd1 enzyme were further reduced (Fig 3B).

The transcription factors Srebp-1 and Lxr are involved in controlling the levels of lipogenic gene enzymes [41]. Therefore, the *Srebp-1* and *Lxrα* expression was examined in mice fed STD or HFD. An increase in the *Srebp-1* expression was observed in animals fed HFD and the treatment with rIL-6 in this last group further increased these levels (S1 Fig). No major changes were observed for *Lxr* expression in any of the nutritional and treatment conditions. These last results indicated that the down-regulated expression of lipogenic enzymes observed after rIL-6 treatment could not be attributed to a negative expression of the Srebp-1 and Lxr factors.

**Fig 4:**
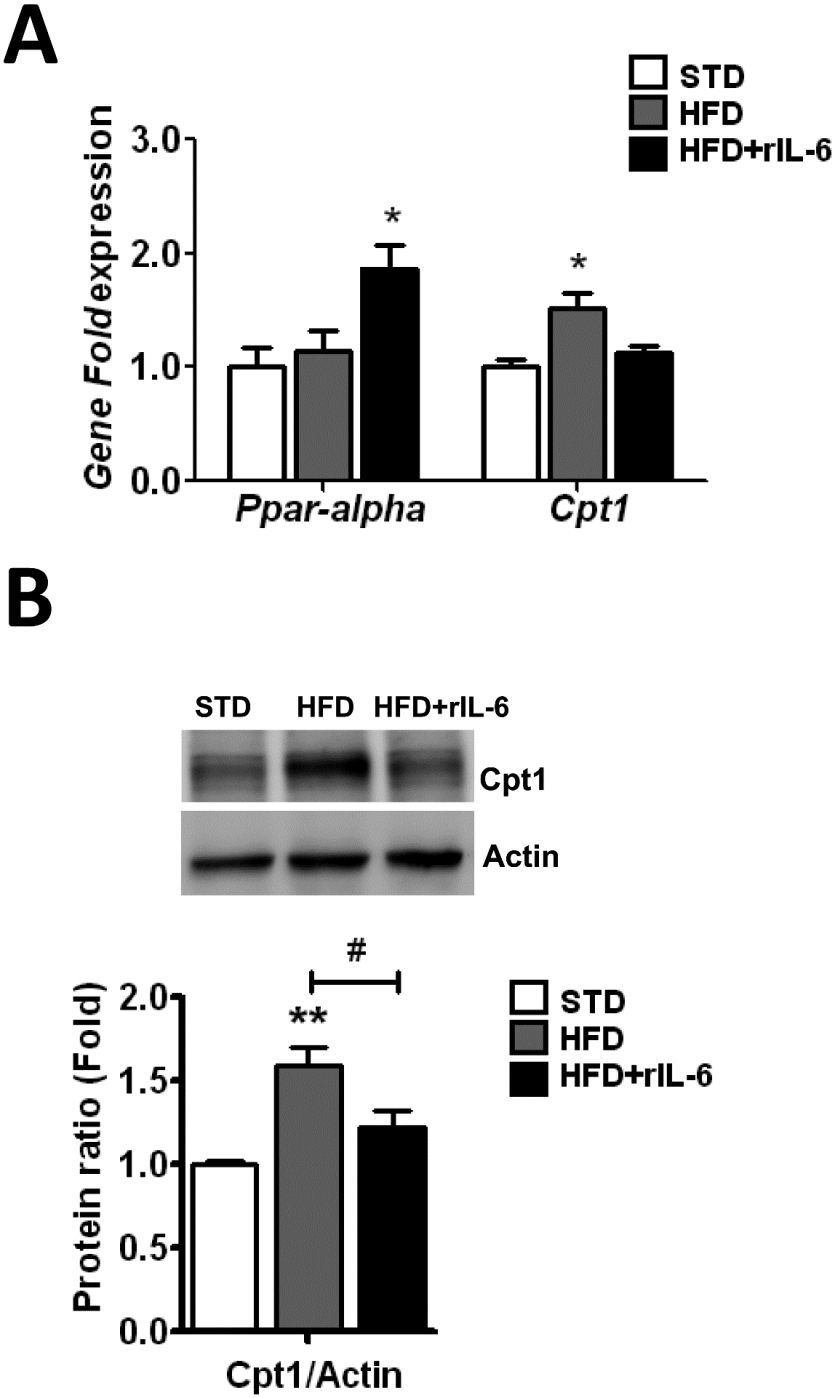
Effect of IL-6 treatment on the expression of *Ppar-alpha* and its target gene *Cpt1* in the livers of WT mice fed a HFD. The qPCR analysis for the gene expression of *Ppar-alpha* and *Cpt1* **(A)** in the livers of mice fed STD, HFD or HFD+rIL-6 is depicted in the histogram. The expression of each gene was normalized using Biogazelle’s qBase^PLUS^ software with *Gapdh* and *Gus-beta* as reference genes. The columns represent CNRQ means ± SEM (n = 8 animals per group). (**B**) Western blot analysis of Cpt1 protein expression in liver of mice fed a STD, HFD or HFD+rIL-6 is shown in the upper panel. Representative blots of each protein from one sample out of eight per group are presented. The corresponding expression of actin is shown as a loading control per lane. The histogram below the blot depicts the levels of Cpt1 determined through densitometry corrected for actin. Comparisons between diet conditions were analyzed using one-way ANOVA. *P<0.05, **P<0.01 denote significant differences compared with the corresponding STD-fed group. ^#^*P*<0.05 denotes significant differences in expression between HFD-fed and HFD+rIL-6 groups.

IL-6 treatment *in vivo* increases the expression of the hepatic transcription factor Ppar-alpha which regulates the expression of Cpt1 gene enzyme, which in turn is involved in the beta-oxidation of fatty acids [10, 18, 42]. To further understand the molecular mechanisms underlying the IL-6 aggravation of fatty liver we also examined the *Ppar-alpha* expression and the expression of its target gene *Cpt1*. Compared with the group fed a STD, no changes were observed for *Ppar*-*alpha* expression profile after HFD exposure, though the subsequent treatment with rIL-6 up-regulated (*P*<0.05) its expression (Fig 4A). The *Cpt1* expression showed a significant (*P*<0.05) increase after a HFD but it was not increased after treatment with rIL-6 (Fig 4A). Likewise, analysis of the protein amounts revealed that Cpt1 was increased in the HFD group but not in the rIL-6-treated HFD group (Fig 4B). Contrary to what was expected, these last observations indicate that most probably the activity of Ppar-alpha was repressed after the chronic treatment with rIL-6.

### 3. Chronic administration of rIL-6 affects endogenous *IL-6* expression negatively and increases *Tnf-alpha* expression in liver of DIO mice

**Fig 5:**
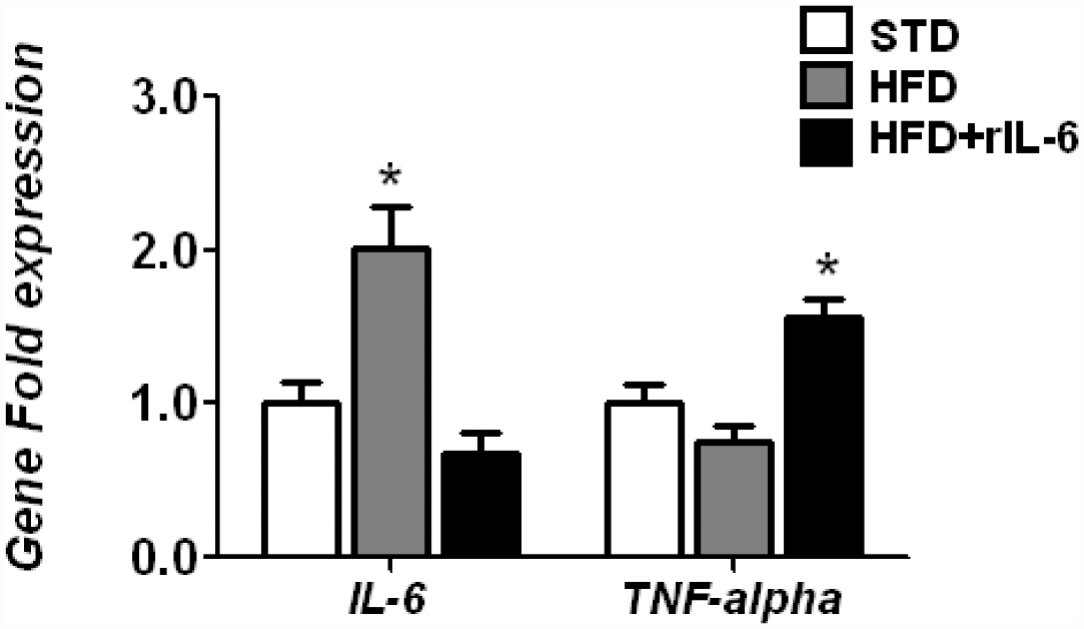
Effect of chronic administration of IL-6 on the hepatic expression of *IL-6* and *Tnf-alpha* in the livers of WT mice fed a HFD. Endogenous expression of the genes *IL-6* and *Tnf-alpha* in liver samples from mice fed STD, HFD or HFD+rIL-6, determined through qPCR analysis. Normalization was performed by Biogazelle’s qBase^PLUS^ software with *Gapdh* and *Gus-beta* as reference genes. The columns represent CNRQ means ± SEM (n = 8 animals per group) and the significance of differences between groups was analyzed by one-way ANOVA. *P<0.05 denotes significant differences compared with the corresponding STD-fed group.

Recent studies showed that IL-6^-/-^ mice exhibited signs of liver inflammation [5], and when the IL-6R was deleted only in hepatocytes, mice developed liver inflammation, which could be reduced by Tnf-alpha blockade, suggesting a pivotal balance of IL-6 and Tnf-alpha signalling in the liver [43]. Previously we also determined that the chronic administration of rIL-6 was able to inhibit the up-regulation of *Tnf-alpha* found in liver of IL-6^-/-^ mice fed a HFD [12]. Following this, we analyzed the effects of IL-6 administration on the modulation of the gene expression of the endogenous *IL-6* and *Tnf-alpha* in the liver of WT mice subjected to DIO. In agreement with previous studies [12], DIO mice showed an increased expression of endogenous *IL-6* but no major changes in *Tnf-alpha* levels (Fig 5). However, the subsequent chronic administration of rIL-6 significantly reduced the levels of hepatic *IL-6* whereas it up-regulated the levels of *Tnf-alpha* (Fig 5). Thus, the Tnf-alpha may have been produced locally, thereby exerting a restricted response in liver.

### 4. Chronic administration of rIL-6 during a HFD hampers gene up-regulation of the IL-6R/gp130 complex and Stat3, and increases Socs3 amounts in liver

**Fig 6:**
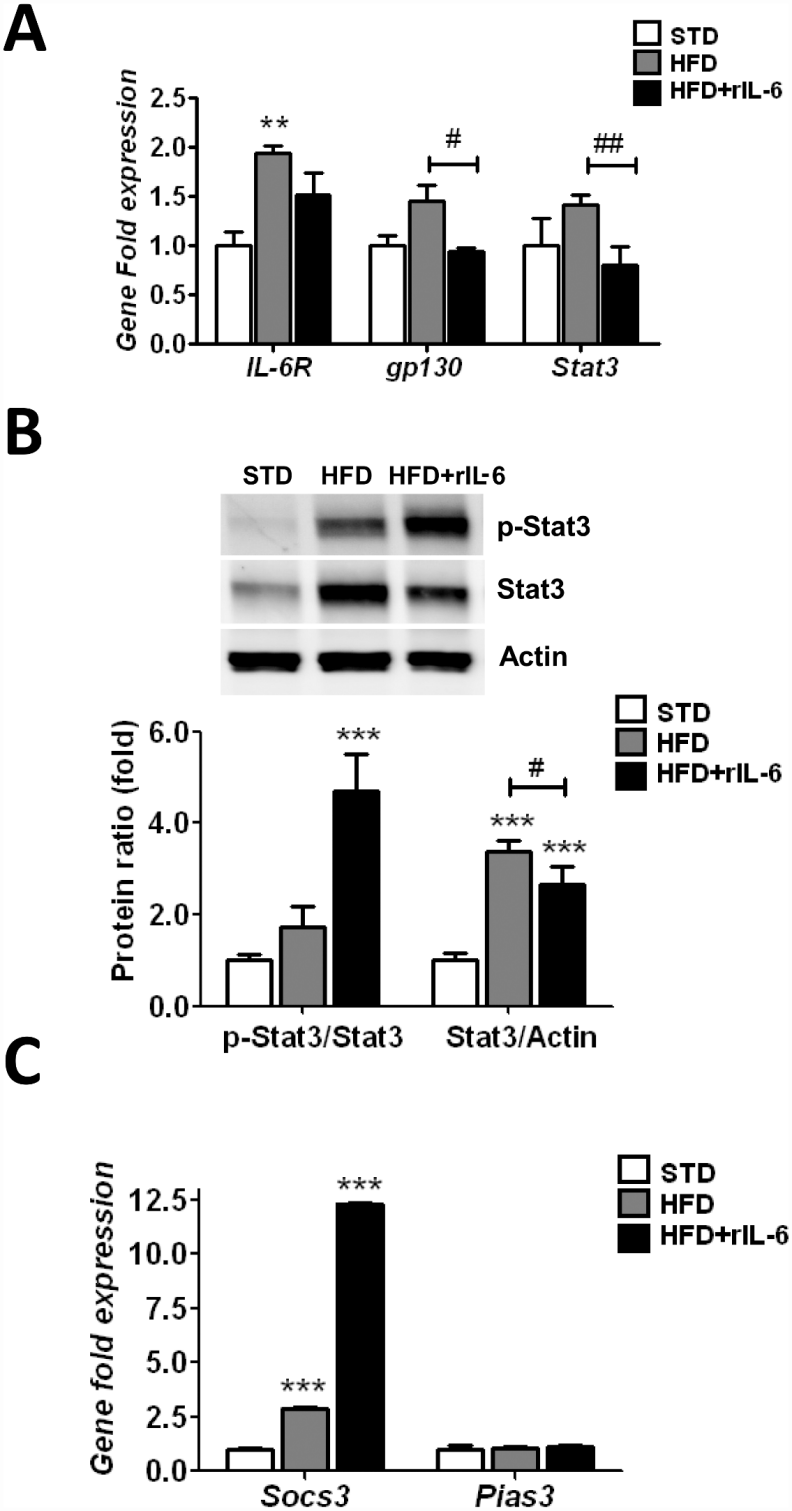
Effect of chronic IL-6 treatment on the expression of components of the IL-6-signalling transduction in liver of WT mice fed a HFD. **(A)** Gene expression of *IL-6R*, *gp130* and *Stat3* in the livers of mice fed STD, HFD or HFD+rIL-6 determined through qPCR analysis. Normalization was done using Biogazelle’s qBase^PLUS^ software with *Gapdh* and *Gus-beta* as reference genes. The columns represent CNRQ means ± SEM (n = 8 animals per group). **(B)** Representative western blot analysis of one sample out of eight of the hepatic expression of Stat3 and p-Stat3 in WT mice fed a STD, HFD or HFD+rIL-6. The corresponding expression of actin is shown as loading control per lane. The histograms below the blots depict the ratios for p-Stat3/Stat3 and Stat3/actin determined through densitometry in each diet condition. The values represent the means ± SEM (n = 8 samples per group). **(C)** Analysis by qPCR of *Socs3* and *Pias3* gene expression in livers of mice fed a STD, HFD or HFD+rIL-6. Normalization was done using Biogazelle’s qBase^PLUS^ software with *Gapdh* and *Gus-beta* as reference genes. The columns represent CNRQ means ± SEM (n = 8 animals per group). For A, B and C the significance of differences between diet groups was evaluated using one-way ANOVA and Bonferroni *post-hoc* tests. *P<0.05, **P<0.01 and ***P<0.001 denote significant differences in expression compared with the corresponding STD-fed group. ^#^*P*<0.05 and ^##^*P*<0.01 denote significant differences in expression between HFD-fed and HFD+rIL-6 groups.

The results described in the previous sections suggested that IL-6 signalling was likely regulated negatively in the presence of high amounts of rIL-6. This prompted us to analyze the expression and the status of the components of the IL-6-signalling transduction pathway in the conditions assayed. First, we examined the regulation of *IL-6R* and *gp130* gene expression under a STD and a HFD. Feeding a HFD caused a 2-fold increase (*P*<0.05) in hepatic gene expression of IL-6R, an effect that was not evident after IL-6 treatment (Fig 6A). A tendency to an increased *gp130* expression was observed in the HFD-fed group, but after chronic treatment with rIL-6 the levels of *gp130* chain expression returned to baseline levels and a *post hoc* test showed significant (*P*<0.05) differences between the HFD and HFD+rIL-6 groups (Fig 6A).

Stat3, the main component of the IL-6-mediating signalling machinery, has been described as a transcription factor contributing to the up-regulation of the lipogenic enzymes in hepatocytes [19, 34, 36]. Thus, we next assessed the effects of the exogenous administration of IL-6 on the gene expression and activation status of Stat3. A modest 1.4-fold increase (*P*<0.05) in *Stat3* expression was observed in the HFD-fed group with respect the STD-fed group. This up-regulation was not found in the HFD+rIL-6 group, which showed basal levels of *Stat3*. Indeed, a *post hoc* test showed differences (*P*<0.01) between the HFD and HFD+rIL-6 groups (Fig 6A). Likewise, an increase (*P*<0.01) in the ratio of the Stat3 band density to the actin band density was observed in animals fed a HFD; nevertheless, the p-Stat3/Stat3 ratio, representing changes in the phosphorylated form, showed no main differences compared to the control group (Fig 6B). Interestingly, the treatment with rIL-6 hampered the increase in the Stat3/actin ratio even though the p-Stat3/Stat3 ratio was significantly enhanced (*P*<0.001), thus indicating that the treatment with rIL-6 resulted in the increased activation of Stat3 (Fig 6B).

We next examined at gene and protein levels the regulation of Socs3 and Pias3, two potent negative regulators of IL-6-signal transduction [20]. In the HFD-fed mice, the levels of hepatic *Socs3* were up-regulated 3-fold (*P*<0.05) compared with the levels found in the STD-fed group (Fig 6C). The administration of exogenous IL-6 in HFD-fed mice increased the liver expression of *Socs3* 12-fold (*P*<0.001) (Fig 6C). Analysis of the *Pias3* expression showed no major variations in the expression levels in any of the diet conditions assayed (Fig 6C).

Although not significant, analysis of the Socs3 protein expression revealed a tendency to lower protein amounts in samples from the group treated with rIL-6 (S2 Fig). This observation indicated that either the mRNA levels of *Socs3* remained stabilized or that the Socs3 protein was being degraded rapidly. Nevertheless, whatever amount of Socs3 was expressed, it was evidently not enough to completely hinder the phosphorylation of Stat3. Likewise, the western blot analysis revealed no significant variations in protein Pias3 levels between groups (S2 Fig).

## Discussion

Non-alcoholic fatty liver disease covers a spectrum of liver diseases, ranging from simple steatosis to non-alcoholic steatohepatitis (NASH) and cirrhosis [44]. In our assay conditions, even though chronic treatment with IL-6 aggravated the steatosis under a HFD, we observed no important inflammatory manifestations (e.g., lymphocyte infiltration) in liver samples. These observations are in agreement with a previous study in which we found that IL-6 replacement in IL-6^-/-^ mice fed a HFD aggravated the steatosis [12]. In that case, the IL-6 administration restored the hepatic Stat3 phosphorylation under a HFD, as well as up-regulating the expression of the lipogenic enzymes and worsening the steatosis [12]. This is in agreement with our recent findings showing that hepatic cells treated with siRNA Stat3 are no longer able to up-regulate lipogenic enzyme genes after IL-6 exposure [36]. Indeed, the depletion of liver Socs3, the negative regulator of Stat3 activation, has been shown to promote hepatic lipogenesis and the development of NAFLD under DIO [13, 38]. Likewise, in the present study, we found that chronic treatment with rIL-6 in WT DIO mice increased hepatic Stat3 phosphorylation and exacerbated steatosis. However, unlike in the IL-6^-/-^ mice, this was concomitant with the drop in lipogenic enzyme expression at both gene and protein levels. Our analysis revealed that the down-regulated expression of lipogenic enzymes after rIL-6 treatment could not be attributed at least to a negative expression of the *Srebp-1* and *Lxr* transcription factors, although a defect in their transcriptional activity cannot be ruled out. Paradoxical IL-6 actions in both WT and IL-6^-/-^ phenotypes have also been observed in previous studies. Indeed, we have previously described the up-regulation of lipogenic enzymes after a single low dose of IL-6 in IL-6^-/-^ mice fed a normal diet but the same effect was not so obvious in the WT mice [36]. Wallenious et al. described the reduction of body weight after treatment with IL-6 in IL-6^-/-^ mice whereas there was no effect on WT mice [13]. These paradoxical actions of IL-6 in WT and IL-6^-/-^ mice probably reflect different mechanisms concerning the modulation of the IL-6-mediated signalling between the two genotypes. In this regard, studies showing that pre-stimulation with IL-6 renders cells less sensitive to further stimulation with IL-6 [45] would support the idea of a refractory response to IL-6 stimulation in the WT HFD-fed mice, which most likely is absent in the IL-6^-/-^ mice, which thereby become more receptive to IL-6 response.

Following the line of reasoning mentioned above we found in this study that: first, the *Cpt1* at gene and protein levels, up-regulated in HFD conditions, was no longer up-regulated after treatment with rIL-6 even though the animals were exposed to a HFD, thus suggesting a failure in IL-6-mediated signalling. Since Cpt1 is involved in the beta-oxidation of fatty acids, its deficit is compatible with exacerbated steatosis. Likewise, the inhibition of the lipogenic enzyme expression shown here after IL-6 administration should, to a certain extent, compensate the HFD-induced steatosis but it also suggests a failure in IL-6-mediated signalling. This is consistent with the increase observed in serum cholesterol levels in mice fed a HFD and treated with rIL-6 and with the tendency to lower serum triglyceride concentrations while increasing triglyceride concentrations in the liver (Fig 1 and Table 1).

Second, the raised levels of circulating leptin found in DIO mice after repetitive administration of exogenous IL-6 suggests the development of leptin resistance. Interestingly, this is indeed one of the characteristics of obese mice lacking IL-6 [13]. The finding that the high leptin levels in WT DIO mice treated with IL-6 were comparable to those found in the IL-6^-/-^ DIO mice (Table 2) is consistent with a desensitization of the IL-6 response. Indeed, the analysis of the LepR revealed a lower phosphorylation status in samples from HFD+rIL-6 group as compared with the phosphorylation levels found in the HFD fed group (Fig 2). In addition, we observed in DIO mice that the IL-6 treatment elevated peripheral glucose levels (Table 2), which is in agreement with other studies in humans showing a correlation between increased levels of IL-6 and increased blood glucose [27]. But again it is surprising that the total depletion of IL-6 in DIO mice seemed also to cause an increase in blood glucose (Table 2), a finding described also in old obese IL-6^-/-^ mice [13]. Therefore, it seems that excess IL-6 causes similar effects to total depletion of IL-6 in obese conditions. Also interesting is the fact that the phenotype observed in DIO WT mice treated with IL-6 is reminiscent of leptin-deficient animals such as the obese Zucker rat model, which shows hyperleptinemia, increased amounts of IL-6 and glucose, and suffers dyslipidaemia, diabetes and steatosis [46], thus suggesting a tight link between both IL-6 and the leptin signalling systems in the liver.

Third, IL-6 exerts an anti-inflammatory function through its inhibitory effects on *Tnf-alpha* gene expression [47, 48]. We previously showed that IL-6^-/-^ mice exposed to a HFD exhibited up-regulation of hepatic *Tnf-alpha* and that chronic rIL-6 treatment lessened the extent of this up-regulation [12]. Paradoxically, in the present study we found that HFD-fed mice exposed to exogenous IL-6 showed a marked *Tnf-alpha* up-regulation. Again, this is compatible with desensitization of the IL-6-mediated signalling pathway. Moreover, the Tnf-alpha response is involved in the stabilization of *Socs3* mRNA [49].

Fourth, the prolonged Stat3 activation observed in the liver of IL-6-treated mice *a priori* argues against efficient negative regulation of IL-6-mediated signalling. Analysis of the negative regulator of IL-6 signalling, Socs3, revealed that after IL-6 treatment in DIO, the mice showed a marked increase in *Socs3* expression while the levels of its protein remained unchanged (Figs 5C, S1). The *Tnf-alpha* up-regulation found in liver of mice treated with rIL-6 would provide an explanation for the discrepancy between high levels of the Socs3 mRNA and the apparent normal levels of its protein [49]. Socs3 binds simultaneously to specific sites of the receptor subunit gp130 and Jak2, thereby hampering Stat3 activation [50]. Shortly thereafter, Socs3 undergoes a precise post-transcriptional regulation, which rapidly induces degradation in the proteasome [37, 50]. Concerning our findings, we cannot exclude the possibility that Socs3 exerts its function before being rapidly degraded. Indeed, as Socs3 is known to contribute to leptin resistance [51], the high levels of leptin found in serum of mice treated with rIL-6 could reflect leptin receptor failure as a result of the Socs3 activity in these mice. It can also be argued that Stat3 rephosphorylation after hours of exposure to IL-6 has been described even in the continued presence of Socs3 [52]. Indeed, rephosphorylation of Stat3 has been suggested to occur because interaction between Socs3 and the IL-6 receptor is somehow prevented [52].

**Fig 7:**
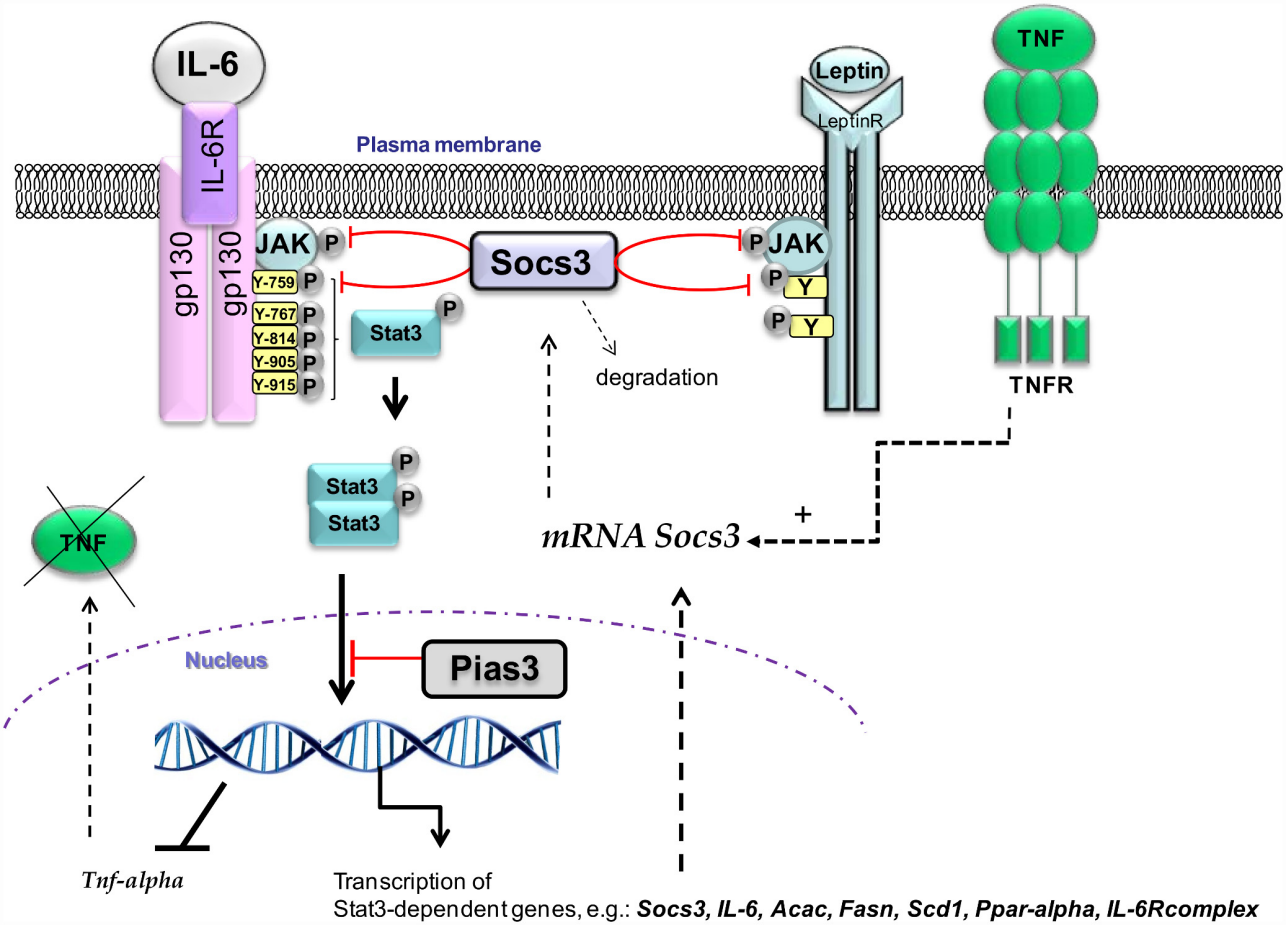
Diagram representing a putative mechanistic model of the desensitizing of the IL-6-mediated signal in liver of DIO mice. IL-6 binds its IL-6R/gp130 receptor complex, resulting in its autophosphorylation at 5 tyrosine residues and the activation of Jak which then induces the recruitment of the Stat3 and its phosphorylation. The phosphorylated Stat3 dimerizes and migrates to the nucleus to initiate transcription of Stat3-dependent genes, e.g., *lipogenic genes*, *IL-6*, *Stat3*, *IL-6R/gp130*, *Ppar-alpha*, and *Socs3*. Induced Socs3 protein is recruited to the phosphotyrosine 759 (Y-759) motif of the activated gp130 and inhibits JAK activity. Nevertheless, as proposed elsewhere, IL-6 may overcome Socs3 inhibition because there are 4 additional tyrosine phosphorylation sites in gp130, distal to the Tyr759 residue, that could allow ligand-mediated Stat3 phosphorylation in vivo [19]. The Pias3 protein, which is normally expressed constitutively, may act like a buffer that titrates the concentration of activated Stat3 dimers within the cell [20] thereby attenuating the Stat3 function. As a result, the transcription of Stat3-dependent genes is suppressed, and subsequently the *Tnf-alpha* gene, which is down-regulated in liver by IL-6, is therefore induced. The locally produced Tnf-alpha can mediate the *Socs3* mRNA stabilization [49]. Although the Socs3 protein can be rapidly degraded [37, 50], it inhibits the leptin response therefore leading to leptin resistance [51]. Altogether, this would lead to the phenotype described in the IL-6^-/-^ DIO mice showing dyslipidaemia, elevated leptin, hyperglycaemia, and aggravated liver steatosis, as occurs in obese leptin-deficient animals [12, 46].

Fifth, the phosphorylation status observed in Stat3 does not agree with either the down-regulated expression of genes depending on IL-6/Stat3-mediated signalling (like those of the lipogenic enzymes) or the up-regulated expression of *Tnf-alpha*. This strongly suggests that even though Stat3 is phosphorylated, the Stat3 DNA binding activity is somehow dampened in our model. Indeed, the transcription of IL-6-dependent genes, like endogenous *IL-6*, *IL-6R/gp130* complex, *Stat3*, *lipogenic enzymes*, and *Cpt1* [12, 35, 53], is down-regulated in the liver of mice fed a HFD and treated with IL-6. A plausible explanation for these observations is the involvement of Pias3, which interacts specifically with the phosphorylated form of the Stat3 molecules after IL-6 stimulation, thereby negatively affecting its transcriptional activity [39]. The constitutive expression of Pias3 shown here is consistent with the notion that its physiological function differs from that of Socs3, which is induced in a negative feedback loop on cytokine stimulation. It has been proposed that Pias3 protein may act like a buffer that titrates the concentration of activated Stat3 dimmers within the cell [20]. Consequently, as outlined in Fig 7, it can be speculated that binding of Pias3 to phosphorylated Stat3 accounts for the inhibited expression of the IL-6-signalling pathway target genes in the liver of DIO mice after chronic IL-6 treatment.

### Conclusions

Overall, our results suggest that both deficiency and excess of IL-6 can lead to similar effects, particularly in the context of fatty liver disease. This paradox is probably the result of adjustments in signal transduction pathways to compensate for the imbalanced concentrations of the IL-6. As proposed elsewhere, whether the IL-6 effects were “good or evil” seems to depend on the site, the time of production, the amounts, and the metabolic context [8]. The present results underscore the importance of the duration of exposure to IL-6, even at low levels, on desensitization of the IL-6 receptor in relation to progression of NAFLD in a WT context. In summary, it is quite possible that the high circulating levels of IL-6 observed in obesity generate, in turn, the down-modulation of the signalling pathway of IL-6 and ultimately a deficit of its signal, producing an IL-6-deficient like situation reminiscent of that found in obese IL-6^-/-^ mice and obese Zucker rat models.

## Supporting Information

S1 Fig Gene expression of *Srebp-1* and *Lxrα* in the livers of WT mice fed HFD and treated with rIL-6. The gene expression of *Srebp-1* and *Lxrα* in the livers of WT fed STD, HFD or HFD-treated chronically with rIL-6 (HFD+rIL-6) is shown in the histogram. The gene expression was determined through qPCR analysis of the liver samples. The expression of each gene was normalized using Biogazelle’s qBase^PLUS^ software with *Gapdh* and *Gus-beta* as reference genes. The columns represent CNRQ means ± SEM (n = 8 animals per group). The significance of differences between groups was evaluated using one-way ANOVA for each gene. *P*<0.05, **P<0.01 denote significant differences compared with the corresponding STD-fed group.
(TIF)

S2 Fig Socs3 and Pias3 expression in liver of HFD-fed mice treated chronically with rIL-6. Representative western blot analysis for Socs3 and Pias3 proteins out of five samples per group (STD, HFD and HFD+rIL-6). The corresponding expression of actin is shown as loading control per lane. The ratios for Socs3/actin and Pias3/actin determined through densitometry are shown in the histogram below. The values represent the means ± SEM. The significance of differences between groups was evaluated using one-way ANOVA.
(TIF)
