# Supplementary figures and images for: Chronic IL-6 Administration Desensitizes IL-6 Response in Liver, Causes Hyperleptinemia and Aggravates Steatosis in Diet-Induced-Obese Mice
Source: PLoS One. 2016 Jun 22;11(6):e0157956. doi: 10.1371/journal.pone.0157956 (PMC4917096; doi:10.1371/journal.pone.0157956)

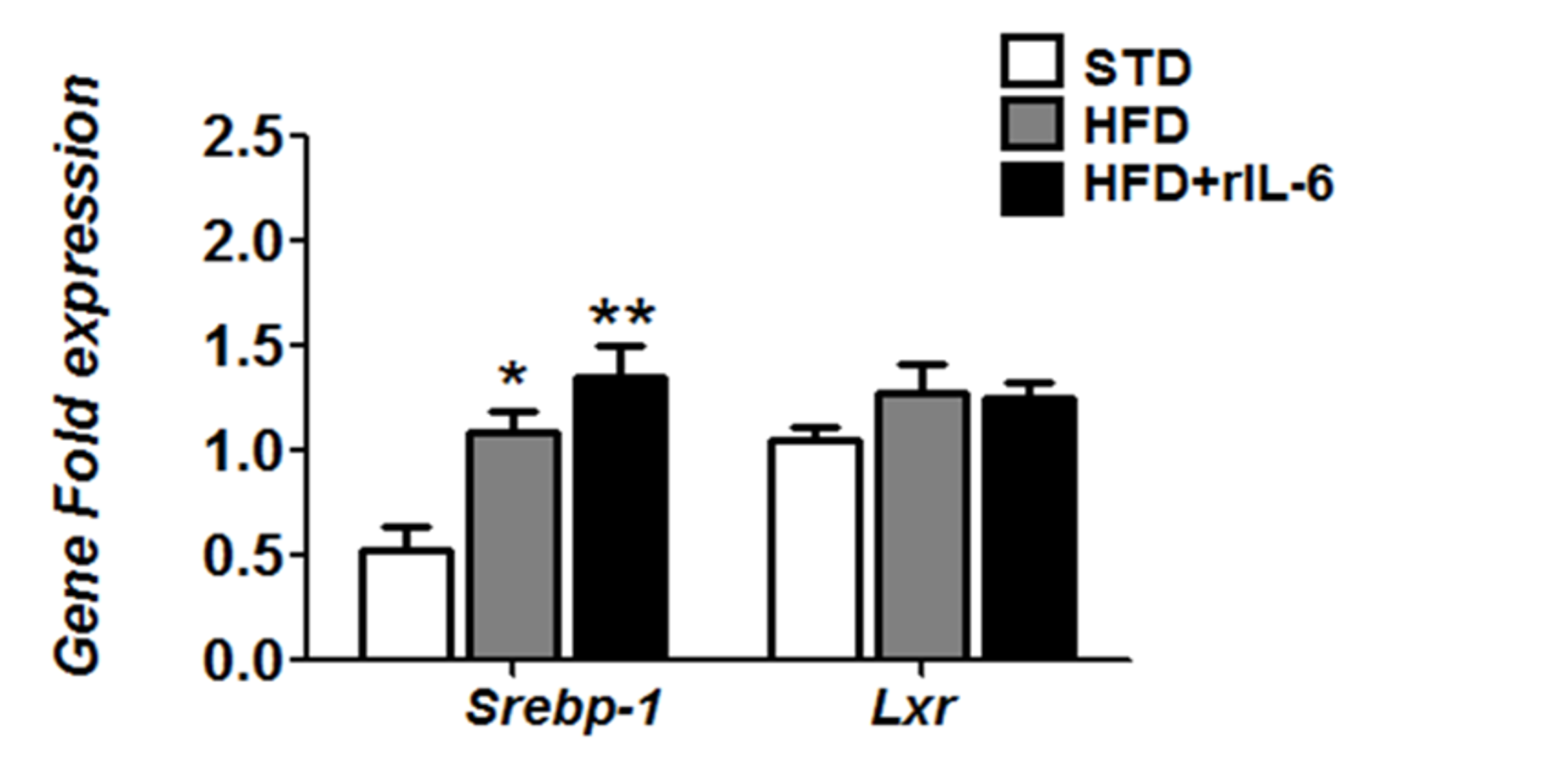

Supplement: S1 Fig — The gene expression of Srebp-1 and Lxrα in the livers of WT fed STD, HFD or HFD-treated chronically with rIL-6 (HFD+rIL-6) is shown in the histogram. The gene expression was determined through qPCR analysis of the liver samples. The expression of each gene was normalized using Biogazelle’s qBasePLUS software with Gapdh and Gus-beta as reference genes. The columns represent CNRQ means ± SEM (n = 8 animals per group). The significance of differences between groups was evaluated using one-way ANOVA for each gene. P<0.05, **P<0.01 denote significant differences compared with the corresponding STD-fed group. (TIF) [file pone.0157956.s001.tif]

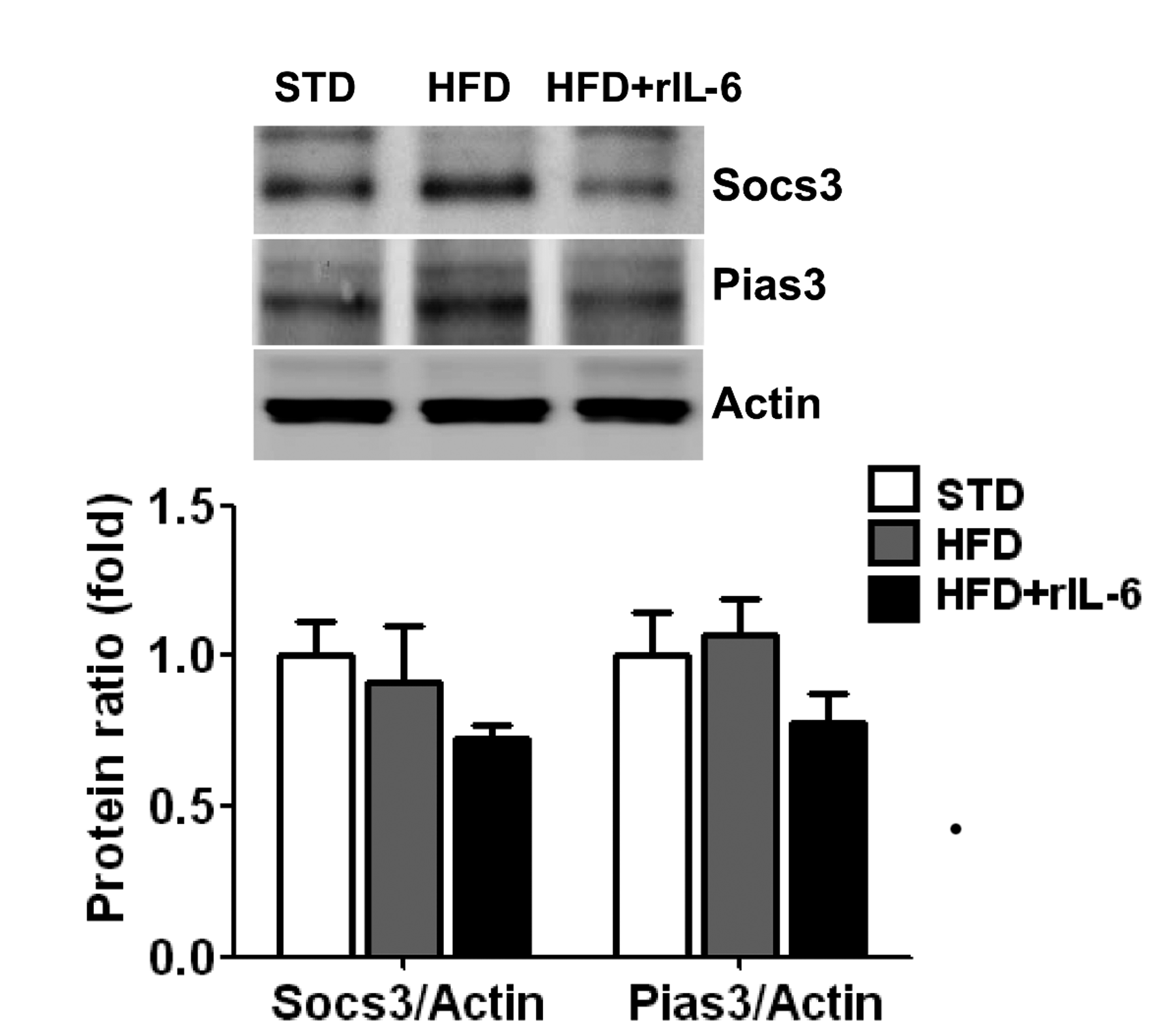

Supplement: S2 Fig — Representative western blot analysis for Socs3 and Pias3 proteins out of five samples per group (STD, HFD and HFD+rIL-6). The corresponding expression of actin is shown as loading control per lane. The ratios for Socs3/actin and Pias3/actin determined through densitometry are shown in the histogram below. The values represent the means ± SEM. The significance of differences between groups was evaluated using one-way ANOVA. (TIF) [file pone.0157956.s002.tif]
